# Editorial: Neural Mechanism for Social Interaction: From Molecules to Neural Circuits

**DOI:** 10.3389/fncir.2022.938354

**Published:** 2022-07-29

**Authors:** Jun Wang, Nan-Jie Xu, Weizhe Hong, Han Xu

**Affiliations:** ^1^Department of Neurobiology and Neurology, The Second Affiliated Hospital, Zhejiang University School of Medicine, Hangzhou, China; ^2^Department of Anatomy and Physiology, Shanghai Jiao Tong University School of Medicine, Shanghai, China; ^3^Department of Biological Chemistry and Neurobiology, David Geffen School of Medicine, University of California, Los Angeles, Los Angeles, CA, United States

**Keywords:** social cognition, social behavior, social dysfunction, neuropsychiatric disorders, neural circuitry

Social interaction accounts for a large amount of our daily life and represents the cornerstone of human society (Chen and Hong, 2018; Matthews and Tye, 2019). Conversely, social impairments are commonly observed in various neurodevelopmental, neuropsychiatric and neurodegenerative disorders, such as depression, autism, and social anxiety disorder (Hari et al., 2015). How our brain controls social interaction behaviors and what pathological substrates underlie social deficits in distinct neuropsychiatric disorders are current Research Topics of intense investigation. Here, we present a Research Topic that contains a collection of both original research articles and review articles, highlighting recent progress in the developmental, neuroanatomical, and molecular mechanisms of social behaviors.

For group living animals, social hierarchy is a general feature and of critical importance for survival, health, and reproduction (Zhou et al., 2018). Social hierarchy is plastic in rodents and is under bidirectional regulation of social experience. For example, previous history of winning experiences increases the probability of victory in subsequent competitions, and this depends on long-lasting plastic changes in the thalamo-prefrontal circuit (Zhou et al., 2017). Interestingly, in this Research Topic, Bicks et al. show that a single losing experience destabilizes hierarchy in adolescent but not in adult mice, suggesting a critical window for experience-dependent plasticity of social dominance hierarchy. Moreover, the development of social hierarchy relies on activity of the prefrontal cortex and is regulated by critical period plasticity in primary sensory cortical areas. This study provides a deeper understanding of the mechanisms underlying formation and maintenance of social hierarchy.

To display social behavior, animals need to instantaneously integrate multiple internal states (e.g., motivation, reward, emotion, and memory) with ever-changing sensory information of different modalities (e.g., visual, auditory, olfactory, and somatosensory). Among these, pheromones serve as an important social cue in rodents (Chen and Hong, 2018). Zhai et al. systematically investigate neuronal inputs to the extraorbital lacrimal glands (ELGs), which are well-known exocrine glands to secrete pheromones (Hirayama et al., 2013). They identify sex-specific differences in the anatomy and physiology of the ELGs. In addition, viral tracing experiments reveal sex-specific differences in their innervations by the axons from the hypothalamus, olfactory areas, and striatum. These valuable observations provide a structural basis for differences in social behaviors between males and females that may involve the ELGs.

Social intelligence represents important abilities to successfully interact with other people in social activities. An fMRI study conducted by Votinov et al. provides new evidence for brain organization underlying social intelligence. They find that the caudate nuclei and the theory-of-mind-related regions play an important role in the maintenance of social intelligence. The level of social intelligence is positively linked to the gray matter volume of the caudate. Moreover, they discuss the potential relevance of these findings to the pathogenesis of mental conditions, such as autism spectrum disorder.

Two articles provide an in-depth review of social interaction in substance use disorder (SUD) and depression, contributed by Beacher et al. and Cahill et al., respectively. These articles summarize the current knowledge in the field but focus on different aspects. The relationship between social interaction and the SUD is poorly understood due to its complexity. On one hand, social withdrawal and isolation are hallmark of SUD. On the other hand, positive social interaction is protective against mental illness of patients with SUD. State-of-the-art methodology is essential to investigate the extraordinarily complicated brain network. The comprehensive review by Beacher et al. provides a thorough review on the application of miniature fluorescence microscope (miniscope) in dissection of neural circuits underlying social interaction and SUD. Miniscopes offer several advantages in studying behavior-related circuits, including the ability to identify specific cell types and ensembles, image at high temporal resolution, target deep brain regions, and monitor neuronal activity in freely moving animals.

Social deficit represents a core behavioral symptom of depression, which is one of the most prevalent mood disorders worldwide. The pathological causes for depression involve complicated environmental and genetic factors. Epigenetics, defined as changes in gene expression without altering the genome sequence, bridges the environmental and genetic mechanism of depression. Cahill et al. summarize major epigenetics modification mechanisms in current literatures and compare epigenetic studies on various rodent models and humans with major depressive disorder. Intriguingly, different epigenetic mechanisms have been linked to several rodent models of depression, such as chronic social defeat stress, chronic variable stress, and early life stress, suggesting diverse epigenetic modulations by a broad range of distinct stressful triggers. Also, depression is a sexually dimorphic disorder, and clinically therapeutic effects vary greatly between men and women. However, neural mechanisms underlying these sex-based differences are not well understood. In this review, Cahill et al. aim to elucidate the sexually dimorphic epigenetic factors controlling social deficits in depression. They discuss sex-specific epigenetic regulations in both depressive animal models and patients. These comprehensive discussions advance our understanding of sexually dimorphic and specific mechanisms for social deficits of depression.

Finally, Wang and Zhan provide a theoretical perspective on social recognition memory, the capacity to allow animals recognize and remember their social partners. Since hippocampus is a brain region well-known for learning and memory, they first focus on hippocampal circuits involved in regulation of social recognition memory. The authors then document the circuit mechanisms underlying how the memory was affected by social isolation. They also extensively discuss cell types and molecules that participate in hippocampal regulation of social recognition memory.

In summary, this Research Topic presents a collection of 6 articles that provide new insights and perspectives into the mechanisms of social interactions and their dysregulations in mental disorders. Moreover, the neuronal and molecular components discussed in these articles may serve as therapeutic targets for social deficits in mental disorders.

## Author Contributions

All authors listed have made a substantial, direct, and intellectual contribution to the work and approved it for publication.

## Funding

This work was supported by grants from the National Natural Science Foundation of China (32125018, 32071005, 31900729, and 32171079), the Fundamental Research Funds for the Central Universities and the MOE Frontiers Science Center for Brain Science & Brain-Machine Integration, Zhejiang University.

## Conflict of Interest

The authors declare that the research was conducted in the absence of any commercial or financial relationships that could be construed as a potential conflict of interest.

## Publisher's Note

All claims expressed in this article are solely those of the authors and do not necessarily represent those of their affiliated organizations, or those of the publisher, the editors and the reviewers. Any product that may be evaluated in this article, or claim that may be made by its manufacturer, is not guaranteed or endorsed by the publisher.
